# High weekly integral dose and larger fraction size increase risk of fatigue and worsening of functional outcomes following radiotherapy for localized prostate cancer

**DOI:** 10.3389/fonc.2022.937934

**Published:** 2022-10-26

**Authors:** Nuradh Joseph, Alessandro Cicchetti, Alan McWilliam, Adam Webb, Petra Seibold, Claudio Fiorino, Cesare Cozzarini, Liv Veldeman, Renée Bultijnck, Valérie Fonteyne, Christopher J. Talbot, Paul R. Symonds, Kerstie Johnson, Tim Rattay, Maarten Lambrecht, Karin Haustermans, Gert De Meerleer, Rebecca M. Elliott, Elena Sperk, Carsten Herskind, Marlon Veldwijk, Barbara Avuzzi, Tommaso Giandini, Riccardo Valdagni, David Azria, Marie-Pierre Farcy Jacquet, Marie Charissoux, Ana Vega, Miguel E. Aguado-Barrera, Antonio Gómez-Caamaño, Pierfrancesco Franco, Elisabetta Garibaldi, Giuseppe Girelli, Cinzia Iotti, Vittotorio Vavassori, Jenny Chang-Claude, Catharine M. L. West, Tiziana Rancati, Ananya Choudhury

**Affiliations:** ^1^ Department of Clinical Oncology, District General Hambantota, Hambantota, Sri Lanka; ^2^ Sri Lanka Cancer Research Group, Sri Lanka College of Oncologists, Maharagama, Sri Lanka; ^3^ Prostate Cancer Program, Fondazione IRCCS Istituto Nazionale dei Tumori, Milan, Hambantota, Italy; ^4^ Department of Medical Physics, University of Manchester, Manchester, United Kingdom; ^5^ Leicester Cancer Research Centre, Department of Genetics and Genome Biology, University of Leicester, Leicester, United Kingdom; ^6^ Division of Cancer Epidemiology, German Cancer Research Center (DKFZ), Heidelberg, Germany; ^7^ Department of Medical Physics, San Raffaele Scientific Institute - IRCCS, Milan, Italy; ^8^ Department of Radiation Oncology, San Raffaele Scientific Institute - IRCCS, Milan, Italy; ^9^ Department of Human Structure and Repair, Ghent University, Ghent, Belgium; ^10^ Department of Radiation Oncology, Ghent University Hospital, Ghent, Belgium; ^11^ Department of Radiation Oncology, University Hospitals Leuven, Leuven, Belgium; ^12^ Translational Radiobiology Group, Division of Cancer Sciences, University of Manchester, and The Christie NHS Foundation Trust, Manchester, United Kingdom; ^13^ Department of Radiation Oncology, Universitätsmedizin Mannheim, Medical Faculty Mannheim, Heidelberg University, Mannheim, Germany; ^14^ Department of Radiation Oncology 1, Fondazione IRCCS Istituto Nazionale dei Tumori, Milan, Italy; ^15^ Department of Medical Physics, Fondazione IRCCS Istituto Nazionale dei Tumori, Milan, Italy; ^16^ Department of Oncology and Haemato-Oncology, University of Milan, Milan, Italy; ^17^ Department of Radiation Oncology, University Federation of Radiation Oncology, Montpellier Cancer Institute, Univ Montpellier MUSE, Grant INCa_Inserm_DGOS_12553, Inserm U1194, Montpellier, France; ^18^ University Federation of Radiation Oncology of Mediterranean Occitanie, ICG CHU Caremaux, Nîmes, France; ^19^ University Federation of Radiation Oncology of Mediterranean Occitanie, ICM Montpellier, Univ Montpellier, Montpellier, France; ^20^ Fundación Pública Galega de Medicina Xenómica, Grupo de Medicina Xenómica (USC), Santiago de Compostela, Spain; ^21^ Instituto de Investigación Sanitaria de Santiago de Compostela, Santiago de Compostela, Spain; ^22^ Biomedical Network on Rare Diseases (CIBERER), Madrid, Spain; ^23^ Department of Radiation Oncology, Complejo Hospitalario Universitario de Santiago, SERGAS, Santiago de Compostela, Spain; ^24^ Department of Radiation Oncology, Ospedale Regionale U. Parini-AUSL Valle d’Aosta, Aosta, Italy; ^25^ Department of Radiation Oncology, Istituto di Candiolo - Fondazione del Piemonte per l’Oncologia IRCCS, Candiolo, Italy; ^26^ Department of Radiation Oncology, Ospedale ASL9, Ivrea, Italy; ^27^ Department of Radiation Oncology, Azienda USL – IRCCS di Reggio Emilia, Emilia-Romagna, Italy; ^28^ Department of Radiation Oncology, Cliniche Gavazzeni-Humanitas, Bergamo, Italy; ^29^ University Cancer Center Hamburg, University Medical Center Hamburg-Eppendorf, Hamburg, Germany

**Keywords:** prostate cancer, fatigue, radiotherapy - adverse effects, functional loss, integral dose

## Abstract

**Introduction:**

We hypothesized that increasing the pelvic integral dose (ID) and a higher dose per fraction correlate with worsening fatigue and functional outcomes in localized prostate cancer (PCa) patients treated with external beam radiotherapy (EBRT).

**Methods:**

The study design was a retrospective analysis of two prospective observational cohorts, REQUITE (development, n=543) and DUE-01 (validation, n=228). Data were available for comorbidities, medication, androgen deprivation therapy, previous surgeries, smoking, age, and body mass index. The ID was calculated as the product of the mean body dose and body volume. The weekly ID accounted for differences in fractionation. The worsening (end of radiotherapy versus baseline) of European Organisation for Research and Treatment of Cancer EORTC) Quality of Life Questionnaire (QLQ)-C30 scores in physical/role/social functioning and fatigue symptom scales were evaluated, and two outcome measures were defined as worsening in ≥2 (WS2) or ≥3 (WS3) scales, respectively. The weekly ID and clinical risk factors were tested in multivariable logistic regression analysis.

**Results:**

In REQUITE, WS2 was seen in 28% and WS3 in 16% of patients. The median weekly ID was 13.1 L·Gy/week [interquartile (IQ) range 10.2-19.3]. The weekly ID, diabetes, the use of intensity-modulated radiotherapy, and the dose per fraction were significantly associated with WS2 [AUC (area under the receiver operating characteristics curve) =0.59; 95% CI 0.55-0.63] and WS3 (AUC=0.60; 95% CI 0.55-0.64). The prevalence of WS2 (15.3%) and WS3 (6.1%) was lower in DUE-01, but the median weekly ID was higher (15.8 L·Gy/week; IQ range 13.2-19.3). The model for WS2 was validated with reduced discrimination (AUC=0.52 95% CI 0.47-0.61), The AUC for WS3 was 0.58,

**Conclusion:**

Increasing the weekly ID and the dose per fraction lead to the worsening of fatigue and functional outcomes in patients with localized PCa treated with EBRT.

## Introduction

Radiotherapy-related fatigue is often underestimated in clinical practice in spite of its high prevalence and negative impact on the quality of life (1). The US National Comprehensive Cancer Network defines fatigue as “a distressing, persistent, subjective sense of physical, emotional, and/or cognitive tiredness or exhaustion related to cancer or its treatment that is not proportional to recent activity and interferes with usual functioning” (2). The impact on usual functioning is important. Studies showed increased fatigue and functional deterioration in men with prostate cancer (PCa) treated with radiotherapy, which peaks after the completion of treatment (3, 4). Lower physical activity, poor sleep, depressive mood, and the use of androgen deprivation therapy increase the risk of fatigue (5–7).

The use of whole-pelvis irradiation, to include the draining lymph nodes in the target volume, increases fatigue levels compared with prostate-only radiotherapy, suggesting a plausible correlation between the total body dose, the volume irradiated, and worsening fatigue (8). The integral dose (ID) describes energy deposition within the whole body. Historically, it is considered to measure the “physical aggressiveness” of radiotherapy (9). Modern radiotherapy techniques such as intensity-modulated radiotherapy (IMRT) and volumetric-modulated arc therapy (VMAT) deliver higher doses to the clinical target volume by redistributing the dose away from identified organs at risk, which increases the volume of tissue receiving lower doses of radiation and the ID. No one has explored whether variations in the ID with prostate-only radiotherapy affect the risk of fatigue.

There also have been no studies on the relationship between the dose per fraction and fatigue. Moderately hypofractionated regimens show equivalence with more protracted courses of conventional radiotherapy in localized PCa (10–12). Advances in imaging enabled greater anatomic precision in defining clinical target volumes and reducing motion uncertainties during treatment delivery. This has led to a number of ultrahypofractionated schedules being tested in clinical trials. One such study was the Prostate Advances in Comparative Evidence (PACE-B) trial, which randomized patients to stereotactic body radiation therapy (SBRT) (36.25 Gy in 5 fractions) versus conventional (78 Gy in 39 fractions) or hypofractionated radiotherapy (62 Gy in 20 fractions). Although overall toxicity was similar between the two arms, fatigue was greater in the SBRT arm (overall: 74.5% vs. 57.5%; grade 2 or more: 8.2% vs. 3.2%) (13). These results suggest that radiotherapy-induced fatigue could be higher with larger doses per fraction.

We hypothesized that increasing the pelvic ID and a higher dose per fraction correlate with worsening fatigue and functional outcomes at the end of treatment in localized PCa patients treated with radical radiotherapy without the irradiation of pelvic lymph nodes (14–16).

## Patients and methods

### Patients

The study design was a retrospective analysis of patients recruited into two prospective observational studies. The multinational REQUITE (ISRCTN98496463) and Italian DUE-01 studies were approved by local ethics committees (14–19). PCa patients were enrolled in REQUITE between April 2014 and October 2016 and in DUE-01 between April 2010 and December 2014. Inclusion criteria were patients with localized PCa and definitive treatment with external beam radiotherapy. Exclusion criteria were radiotherapy to the pelvic lymph nodes, the use of a brachytherapy boost, and prior radical prostatectomy.

### Data collection

The following clinical variables were collected prospectively for each patient using standardized forms: age, body mass index, the presence of comorbidities, medication history, previous abdominal and pelvic surgeries, previous transurethral resection of prostate, the use of neoadjuvant androgen deprivation therapy, and treatment technique [IMRT/VMAT versus three-dimensional conformal radiotherapy (3DCRT)]. Radiotherapy planning data were uploaded to VODCA (MSS Medical Software Solutions, Hagendorn, Switzerland). As patients received different fractionation regimens including hypofractionated schedules, the weekly ID was calculated. The ID is the product of the mass of tissue irradiated and the absorbed dose. Although the ID can be calculated with the mass obtained from CT numbers that indicate the distribution of tissue densities in the pelvic region, the approach requires an extra step that potentially misrepresents structures with highly heterogeneous densities (20). Therefore, the differential dose volume histogram (DVH) of the body was calculated in VODCA (MSS Medical Software), and the ID was calculated by multiplying the mean total body dose by the body volume as measured in the radiotherapy treatment planning CT. The weekly ID was computed by dividing the ID by the duration of radiotherapy in weeks.

### Outcome

The quality of life was assessed using European Organisation for Research and Treatment of Cancer Quality of Life Questionnaire-C30 version 3.0 questionnaires, which were completed by patients before radiotherapy and at the following time points: the end of radiotherapy and annually for 2 years in REQUITE and biannually until 5 years in DUE-01 (21). The European Organisation for Research and Treatment of Cancer Quality of Life Questionnaire-C30 comprises distinct multi-item scales and individual items, each of which represents a different aspect of the quality of life. There are five functioning scales (physical, role, social, cognitive, and emotional), three multi-item symptom scales (fatigue, nausea and vomiting, and pain) and six single-item symptoms (dyspnea, insomnia, appetite loss, constipation, diarrhea, and financial difficulties). Apart from the fatigue symptom scale (three items), the physical (five items), role (two items), and social (two items), function scales also assess the quality-of-life aspects related to fatigue. For present analysis, we considered the four above-mentioned multi-item scales, i.e., a total of 12 items.

Differences were measured between the scores in individual scales (scores ranging from 0 to 100) at the completion of radiotherapy vs. the baseline (before the start of radiotherapy). For modeling purposes, we considered “worsening in a specific scale” as a minimum deterioration of 17 points. This is greater than the minimally important differences for within-group deterioration in all the considered scales as reported in studies considering different cancer cohorts (22, 23). Further, this magnitude of change in scores was associated with a large deterioration for all the selected scales, with the exception of role functioning (for this scale, the worsening of 17 points was associated with medium deterioration) (24). Outcome measures were defined as worsening in at least two scales (WS2) and in three out four scales (WS3), possibly identifying the mild and moderate impacts of radiotherapy on everyday life, respectively. Details on the European Organisation for Research and Treatment of Cancer Quality of Life Questionnaire-C30 scales used for this work are reported in **Supplementary Material (Table S1)**.

### Analysis

Univariable logistic regression was used to identify associations between the worsening of functional outcomes (WS2 and WS3) and the following variables: weekly ID, the presence of comorbidities, the use of androgen deprivation therapy, previous surgery, smoking, alcohol, age, body mass index, and radiotherapy delivery (IMRT/VMAT or 3DCRT). Multivariable logistic analysis included covariates from the univariable analysis with p<0.15. Odds ratios (ORs) and 95% confidence intervals (CIs) were used to show the effect size. Models were developed using the REQUITE cohort (TRIPOD 2a model) with DUE-01 used for external independent validation (TRIPOD 3 model) (25). Models were assessed for the goodness-of-fit using the Hosmer–Lemeshow (HL) test, calibration using calibration plots (calibration-in-the-large, calibration slope, and R^2^), and discrimination through the area under the receiver operating characteristics curve (AUC). Internal validation was considered using bootstrapping (1,000 resamples). All statistical analyses were performed using KNIME software (KNIME GmbH, Germany) coupled with R software (www.r-project.org).

## Results

**Supplementary Figures S1**, **S2** show the selection of patients for the REQUITE and DUE-01 populations, respectively. The application of inclusion and exclusion criteria, coupled to the availability of European Organisation for Research and Treatment of Cancer Quality of Life Questionnaire-C30 questionnaires and DICOM data, led to 771 patients: 543 from REQUITE and 228 from DUE-01. **Table 1** lists the clinical and dosimetric characteristics of each cohort.

**Table 1:** Baseline characteristics of the development and validation cohorts.

Patients	REQUITE (n=543)	DUE 01 (n=227)	p-value
Age (years), median and IQ range	72 (67–76)	70.0 (68-75)	0.32
*T-stage*			0.27
T1	36%	61%	
T2a	19%	19%	
T2b	17%	8%	
T2c	13%	10%	
T3a	11%	1%	
T3b	4%	<1%	
T4	1%	0%	
*Gleason score*			0.0001
<7	23%	52%	
3+4	38%	32%	
4+3	21%	9%	
>7	17%	6%	
*pre-RT PSA*			0.001
<10 ng/ml	57%	84%	
10-20 ng/ml	30%	14%	
>20 ng/ml	13%	2%	
*Risk Class*			0.0001
Low	12%	38%	
Intermediate	58%	48%	
High	31%	14%	
*RT Dose*			
Prescribed dose at 2 Gy/fr	76 (74-78) Gy	78 (76-78) Gy	0.001
	[300 pts]	[98 pts]	
Prescribed dose at 2-2.7 Gy/fr	75 (65-77) Gy	70 (70-71.4) Gy	0.35
	[91 pts]	[125 pts]	
in EQD2Gy (α/β = 10 Gy)	77.2 Gy	72 Gy	0.04
in EQD2Gy (α/β = 3 Gy)	80.2 Gy	74.9 Gy	0.03
Prescribed dose at >2.7 Gy/fr	60 (59.4-60.2) Gy	70.2 Gy	0.009
	[152 pts]	[4 pts]	
in EQD2Gy (α/β = 10 Gy)	65.0 Gy	76 Gy	0.61
in EQD2Gy (α/β = 3 Gy)	72.0 Gy	84.2 Gy	0.003
Weekly integral dose (L ∗ Gy/week)	13.1 (10.2-19.3)	15.8 (13.2-19.3)	0.001
*Irradiation technique*			0.0001
3DCRT	17%	15%	
SF-IMRT	13%	39%	
VMAT	70%	46%	
*Hormone therapy*			0.3
No hormone therapy	27%	58%	
Antiandrogen therapy alone	4%	8%	
GnRH analogs alone	40%	24%	
Combined androgen blockade	29%	12%	

IQ, interquartile; RT, radiotherapy; PSA, prostate-specific antigen; EQD2Gy, equivalent uniform dose at 2 Gy/fraction calculated using the linear quadratic model; 3DCRT, three-dimensional conformal radiation therapy; GnRH, gonadotropin-releasing hormone, SF-IMRT, static-field intensity-modulated radiation therapy; VMAT, volumetric-modulated arc therapy.

The p-values for comparison of the clinic–pathological/dosimetric variables of the two cohorts are reported. For continuous features, the t-test was considered, while, for categorical variables, the chi-square test was used.

### Development cohort

In the REQUITE development cohort, the rate of worsening for fatigue, physical functioning, role functioning, and social functioning were 28%, 15%, 30%, and 29%, respectively. Worsening in at least two scales (WS2) was documented in 28% (155/543) of patients, while 16% (85/543) of patients had worsening in three or four scales (WS3). The median weekly ID was 13.1 L·Gy/week [interquartile (IQ) range 10.2-19.3 L·Gy/week]. The proportions with WS2 were 24% for patients below and 33% for those above the median weekly ID. The respective proportions for WS3 were 13% and 18%. The mean weekly IDs were 13.9 L·Gy/week for patients without WS2 and 16.1 L·Gy/week for those with WS2 (t-test p<0.01). The respective values for WS3 were 14.1 L·Gy/week and 16.6 L·Gy/week (t-test p<0.01).

**Figure 1:**
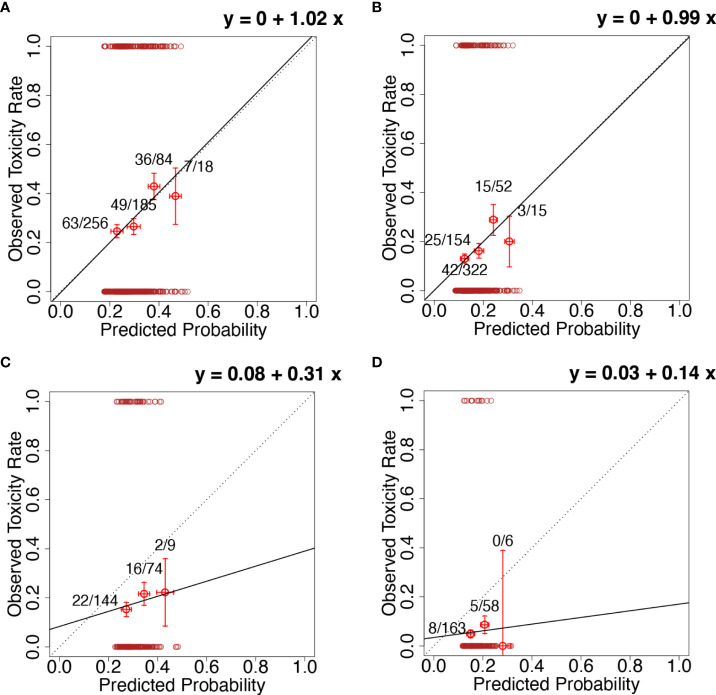
Calibration plots for **(A)** the model for the worsening of at least two functional endpoints with the inclusion of the weekly integral dose (ID) evaluated on the whole REQUITE population (development population); **(B)** the model for the worsening of at least three functional endpoints with the inclusion of the weekly ID evaluated on the whole REQUITE population (development population); **(C)** the model for the worsening of at least two functional endpoints with the inclusion of the weekly ID evaluated on the whole DUE-01 population (independent external validation population); **(D)** the model for the worsening of at least three functional endpoints with the inclusion of the weekly ID evaluated on the whole DUE-01 population (independent external validation population). Calibration plots present the rate of observed events in a group of patients (y-axis) vs. mean predicted probability for the same group (x-axis). Groups of patients are ordered for increasing predicted probability. Error bars represent the confidence interval in observed frequencies as calculated from proportions in the study population and based on the normal distribution of events. The continuous black line represents the calibration line; its equation is given in each plot (calibration-in-the-large and calibration slope). The dotted line represents the calibration line for perfect calibration (i.e., calibration-in-the-large=0 and calibration slope=1). In all plots, the red circles at y=0 and y=1 are the observed events for each patient plotted at the corresponding predicted probability.

Univariable analyses showed that the weekly ID was significantly associated with both WS2 (OR=1.05, 95% CI 1.02-1.08, p<0.01; AUC=0.59, 95% CI 0.55-0.63, HL test p=0.79) and WS3 (OR=1.06, 95% CI 1.02-1.09, p<0.01; AUC=0.60, 95% CI 0.55-0.64, HL test p=0.79). The calibration plots for WS2 and WS3 are shown in **Figures 1A, B**, respectively. Discrimination power was not affected by optimism, with the AUC after the bootstrapping of 0.59 and 0.60 for WS2 (optimism 0.001) and WS3 (optimism 0.002), respectively. Calibration was affected by optimism, with calibration-in-the-large after the bootstrapping of 0.10 and 0.33 and the slopes of 1.11 and 1.19, for WS2 and WS3, respectively. Internal validation confirmed the effect of the weekly internal dose, with the discrimination power not affected by bootstrapping. However, internal validation also highlighted uncertainties in the estimates of the absolute rate of events (calibration-in-the large) and a possible underfitting of the effect size of the weekly internal dose (calibration slope >1). **Table 2** summarizes the results of univariable analyses. The dose per fraction was associated with WS2 with a large effect size (OR=1.8 for each 1 Gy increase in the dose per fraction, p-value<0.01). **Figures S3**, **S4** show WS2 and WS3 as a function of the dose per fraction. The use of IMRT/VMAT versus 3DCRT was associated with both increased WS2 (OR=2.75, p <0.01) and WS3 (OR=3.7, p<0.01).

**Table 2:** Results of univariate logistic regression for the two selected endpoints: (a) the worsening of at least two functional scales (WS2) and (b) the worsening of at least three functional scales (WS3).

	Worsening of ≥2 functional scales (WS2)		Worsening of ≥3 functional scales (WS3)	
Variables	p-value	Odds Ratio (95%CI)	p-value	Odds Ratio (95%CI)
Age (years, continuous variable)	0.29	0.98 (-0.03,+0.03)	0.30	0.98 (-0.03,+0.03)
Body mass index (kg/m^2^,continuous variable)	0.30	1.01 (-0.02,+0.02)	0.20	1.01 (-0.02,+0.02)
Weight (kg, continuous variable)	0.30	1.01 (-0.02,+0.02)	0.27	1.01 (-0.02,+0.02)
Smoking history (current smoker vs. ex-smoker OR never smoker)	0.18	1.49 (-0.66,+1.18)	0.59	1.22 (-0.63,+1.30)
Alcohol (yes vs. no)	0.55	1.14 (-0.41,+0.62)	0.46	1.23 (-0.53,+0.92)
Use of alpha blockers (yes vs. no)	0.86	0.96 (-0.38,+0.62)	0.51	0.80 (-0.38,+0.74)
Use of antidepressant drugs (yes vs. no)	0.87	0.93 (-0.51,+1.12)	0.57	0.73 (-0.48,+1.39)
Use of beta blockers (yes vs. no)	0.11*	0.68 (-0.26,+0.41)	0.30	0.73 (-0.33,+0.59)
Use of lipid-lowering drugs (yes vs. no)	0.14*	1.33 (-0.41,+0.60)	0.52	1.16 (-0.43,+0.69)
Use of 5-alpha reductase inhibitors (yes vs. no)	0.71	1.26 (-0.88,+2-96)	0.37	1.83 (-1.34,+5.01)
Hypertension (yes vs. no)	0.11*	0.74 (-0.23,+0.33)	0.14*	0.71 (-0.26,+0.41)
Diabetes (yes vs. no)	0.007*	0.44 (-0.20,+0.36)	0.02*	0.36 (-0.21,+0.49)
History of heart disease (yes vs. no)	0.46	0.85 (-0.39,+0.46)	0.13*	0.64 (-0.28,0.50)
Depression (yes vs. no)	0.39	0.70 (-0.39,+0.86)	0.44	0.66 (-0.43,+1.24)
Neo(adjuvant) hormone therapy (Yes/No)	0.99	1.00 (-0.34,+0.52)	0.73	0.91 (-0.39,+0.66)
Prescribed dose (EQD2Gy, alpha/beta=10 Gy, continuous variable)	0.04*	0.98 (-0.02,+0.02)	0.34	0.99 (-0.02,+0.026)
Dose per fraction (Gy, continuous variable)	0.002*	1.80 (-0.55,+0.79)	0.15	1.38 (-0.49,+0.76)
Planning target volume (cm^3^, continuous variable)	0.28	1.00 (0,0)	0.19	1.00 (0,0)
Treatment technique (IMRT/VMAT vs. 3DCRT)	0.006*	2.75 (-1.25,+2.31)	0.0009*	3.70 (-2.20,+5.59)
Weekly integral dose (L·Gy/week, continuous variable)	0.0004*	1.05 (-0.03,+0.03)	0.002*	1.05 (-0.03,+0.04)

EQD2Gy, equivalent uniform dose at 2 Gy/fraction calculated using the linear quadratic model; 3DCRT, three- dimensional conformal radiation therapy; IMRT, intensity-modulated radiation therapy; VMAT, volumetric- modulated arc therapy.

P-values <0.05 are in italics; the p-values of the variables used in the multivariable logistic regression are followed with a star(*).

In multivariable analyses, the factors retaining significance for WS2 were the weekly ID, diabetes (OR=0.47), the use of beta-blockers (OR=0.67), and the radiotherapy technique (IMRT/VMAT vs. 3DCRT, OR=1.92). Different radiotherapy techniques can lead to different weekly IDs due to the low–medium dose bath; **Figure S5** shows the distribution of the weekly ID in the REQUITE cohort stratified by the radiotherapy technique (static field radiotherapy vs. volumetric arc radiotherapy). The multivariable model was also significantly associated with WS2 (p<0.0001 for likelihood ratio test; HL test p=0.77; calibration slope 0.99 and calibration-in-the-large 0). There was a small non-significant increase in the AUC (0.63) for the multivariable compared with the univariable model that only included the weekly ID (AUC 0.59). The factors significant for WS3 in multivariable regression were the weekly ID (OR=1.04), diabetes (OR=0.38), and the radiotherapy technique (3DCRT vs. IMRT/VMAT, OR=2.44) (p=0.002 for likelihood ratio test, HL test p=0.65, calibration slope 0.97 and calibration-in-the-large 0). Again, there was a small non-significant increase in the AUC (0.63) for the multivariable compared with the univariable model that only included the weekly ID (AUC 0.60). **Table 3** summarizes the results of multivariable analysis.

**Table 3:** Multivariate model for worsening of at least two and three functional outcomes.

Variable For WRS2 NTCP Model	Coeff	p-value	Odds Ratio(-95%CI,+95%CI)
Weekly integral dose (L·Gy/week, continuous variable)	0.035	0.03	1.035 (-0.033,+0.034)
Treatment technique (IMRT/VMAT vs. 3DCRT)	0.65	0.05	1.92 (-0.97,+1.95)
Diabetes (yes vs. no)	-0.75	0.02	0.47 (-0.22,0.40)
Use of beta blockers (yes vs. no)	-0.40	0.11	0.67 (-0.26,0.42)
Constant	-1.83		
Variable For WRS3 NTCP Model	Coeff	p-value	Odds Ratio (-95%CI,+95%CI)
Weekly integral dose (L·Gy/week, continuous variable)	0.036	0.07	1.037 (-0.033,+0.034)
Treatment technique (IMRT/VMAT vs. 3DCRT)	0.89	0.08	2.44 (-1.56,+4.28)
Diabetes (yes vs. no)	-0.98	0.03	0.38 (-0.22,0.52)
Constant	-2.92		

**Figure 2:**
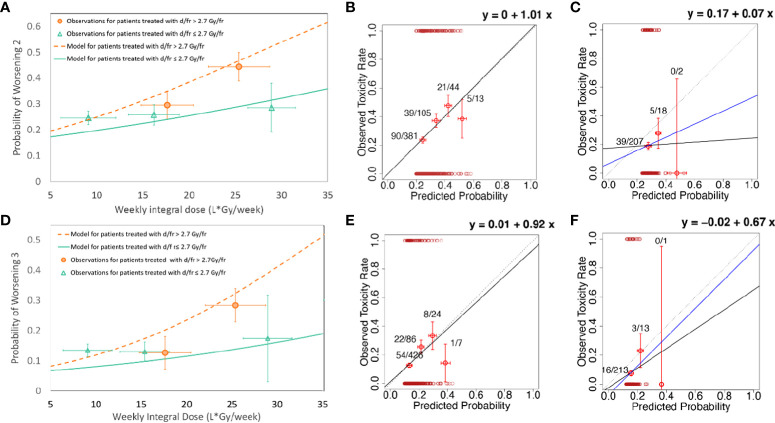
**(A)** Model for the worsening of at least two functional endpoints (WS2) with the inclusion of the weekly ID and stratification for the daily dose; **(B)** the calibration plot for the model for the worsening of at least two functional endpoints (WS2) with inclusion of the weekly ID and stratification for the daily dose evaluated on the whole REQUITE population (development population); **(C)** the calibration plot for the model for the worsening of at least two functional endpoints (WS2) with the inclusion of weekly ID and stratification for the daily dose evaluated on the whole DUE-01 population (independent external validation population); **(D)** the model for the worsening of at least three functional endpoints (WS3) with the inclusion of weekly ID and stratification for the daily dose; **(E)** the calibration plot for the model for the worsening of at least three functional endpoints (WS3) with the inclusion of the weekly ID and stratification for the daily dose evaluated on the whole REQUITE population (development population); **(F)** the calibration plot for the model for the worsening of at least three functional endpoints (WS3) with the inclusion of the weekly ID and stratification for the daily dose evaluated on the whole DUE-01 population (independent external validation population). The continuous black line represents the calibration line; its equation is given in each plot (calibration-in-the-large and calibration slope). The dotted line represents the calibration line for perfect calibration (i.e., calibration-in-the-large=0 and calibration slope=1). In **(C, F)**, the blue line represents calibration after the exclusion of the outlier patients with predicted high risk and no worsening of functional endpoints; see the main text for details.

Due to the high impact of the dose per fraction, a stratified logistic model was fitted separately for patients with a dose per fraction ≤2.7 Gy (98 events out of 391 [25%] patients for WS2; 53/391 events [13.6%] for WS3) vs. a dose per fraction >2.7 Gy (57 events out of 152 [37.5%] patients for WS2; 32/152 [21.1%] events for WS3). Different effect sizes resulted in the two subpopulations for both endpoints: OR=1.03 vs. OR=1.07 for WS2 and OR=1.04 vs. OR=1.09 for WS3 (**Table S2**). **Figure 2** shows the logistic curves for the stratified models for WS2 and WS3 (**Figures 2A, D**) and the corresponding calibration plots (**Figures 2B, E**). The AUC was 0.60 (95% CI 0.55-0.63) for WS2 and 0.61 for WS3 (95% CI 0.57-0.65). Internal validation resulted in an AUC=0.60 (optimism 0.001) and a highly improved stability of calibration when compared with the model not stratified for the dose per fraction. Calibration slopes after bootstrapping were 0.99 and 1.07 for WS2 and WS3, respectively, and calibration-in-the-large was -0.01 (WS2) and 0.10 (WS3).

### Validation cohort

In DUE-01, the rates of the worsening of physical functioning, role functioning, fatigue, and social functioning were 10%, 21%, 20%, and 30%, respectively. WS2 and WS3 proportions were 19.4%, and 8.4%, respectively. The median weekly ID was 15.8 L·Gy/week (IQ range 13.2-19.3 L·Gy/week). The proportions with WS2 were 18.4% for patients below the median weekly ID and 20.4% for those above the median weekly ID. The respective proportions for WS3 were 7.0% and 9.7%. The mean weekly IDs were 16.6 L·Gy/week for patients without WS2 and 16.8 L·Gy/week for those with WS2 (t-test p=0.8). The respective values for WS3 were 16.5 L·Gy/week and 17.9 L·Gy/week (t-test p=0.3). Although the weekly IDs were higher in DUE-01 compared with REQUITE, there was a lower rate of worsening in the scales tested. In particular, there was a substantially reduced worsening of physical and role functioning (**Figure S6**).

Four models were taken forward for validation in the DUE-01 population, including two models each for WS2 and WS3 without and with stratification for the dose per fraction. Multivariable models including clinical risk factors were not considered for validation due to their small non-significant improvement in model performance. The calibration plots for the models applied to the DUE-01 population are presented in **Figures 1**, **2**: (1) WS2 without stratification for dose per fraction in **Figure 1C**; (2) WS3 without stratification for dose per fraction in **Figure 1D**; (3) WS2 with stratification for the dose per fraction in **Figure 2C**; and (4) WS3 with stratification for the dose per fraction in **Figure 2F**. In all cases, a general increase in the rate of the worsening of the physical/functional status with an increased weekly ID was observed but with a reduced slope compared with the development cohort (calibration slopes ranging from 0.14 to 0.67). Calibration-in-the-large showed offsets ranging from -0.02 to 0.17, reflecting the different rates of the study endpoints in DUE-01.

Discrimination power was systematically lower in DUE-01 than REQUITE, but the difference was not statistically significant. The AUC was 0.52 (95% CI 0.42-0.61) for both models on WS2 (with/without stratification for dose per fraction), while, for models on WS3, the AUC was 0.58, (95%CI 0.43–0.67). Of note, models including the stratification for the dose per fraction behaved in a satisfactory way for most patients and failed for only a very small proportion of patients who were classified at high risk but did not have WS2 (two patients) or WS3 (one patient). Excluding these few inconsistent patients improves the calibration for both WS2 (calibration-in-the-large 0.07 and calibration slope 0.46) and WS3 (calibration-in-the-large -0.07 and calibration slope 1.0).

## Discussion

We showed that 15%–30% of patients experience significant worsening fatigue and functional outcomes following external beam radiotherapy (EBRT) for PCa. Our observation that the weekly whole-body ID is a predictor of fatigue was validated (as increasing rate of worsening of functional outcomes with increasing weekly ID) in an independent cohort. This is the first study linking the whole-body ID with acute fatigue and functional outcomes in PCa patients. Our study also highlights that larger fraction sizes increase the risk of fatigue.

Initial studies showed that IMRT increases IDs due to a larger volume of tissue being irradiated to lower doses, raising concerns that it could increase the risk of developing second malignancies (26–28). However, other studies were contradictory with IMRT plans producing lower IDs (29, 30). We found that patients treated with IMRT/VMAT versus 3DCRT had more early fatigue and worse functioning outcomes in univariable analyses, which suggests that irradiating larger volumes of tissue with low doses contributes to an increased risk of fatigue. Regardless of the treatment technique, our results show that the ID needs to be considered in the delivery of external beam radiotherapy.

We could not investigate the mechanistic links of the correlation between the ID and fatigue in this study. Indeed the molecular mechanisms of radiotherapy-induced fatigue have not been fully elucidated, but studies revealed a link with mitochondrial dysfunction and pro-inflammatory immune dysregulation (31, 32). A study on systemic blood counts in breast cancer patients receiving intraoperative RT either as accelerated partial breast irradiation or as boost before external beam RT found that the volume of irradiation may play a role on the direct toxic effect of radiation on circulating blood cells (33). The associations between irradiated volume and changes in hematological parameters were also reported in a PCa study investigating patients treated with postoperative RT and whole-pelvis RT (34). All these processes would be affected by the weekly ID.

Regarding the counterintuitive findings of lower fatigue rates in diabetic patients (13.5% vs. 18.5% for WRS2 and 2.7% vs. 6.3% for WRS3), we could hypothesize some beneficial effects of antidiabetic drugs aside from insulin resistance and glucose metabolism regulation. Indeed, studies report the anti-inflammatory role of metformin (35, 36) and its impact on chronic pain (37), suggesting that these two mechanisms could explain lower fatigue rates and better functional outcomes in PCa patients with diabetes.

We also found that larger doses per fraction increased the risk of fatigue. Interestingly, fraction size predicted the risk of worsening fatigue and functional outcomes even after accounting for overall treatment time, suggesting that radiotherapy-induced fatigue is sensitive to fractionation. Our finding is consistent with the report from the Hypofractionated Radiotherapy of intermediate risk localised Prostate Cancer (HYPO-RT-PC) trial that showed more pronounced early side-effects with ultrahypofractionated compared with conventionally fractionated radiotherapy (38). While the PACE-B trial reported the similar rates of early side-effects for ultrahypofractionated versus conventionally/moderately hypofractionated radiotherapy, the level of ≥grade 2 fatigue was higher (8.2% vs. 3.2%) (13).

The AUC for models was modest (around 0.60). Despite the widespread use of the AUC in evaluating the performance of models for radiotherapy outcomes, radiotherapy side-effects pose a peculiar challenge for a measure that rewards a clear separation of responders from non-responders. The shallow dose–response, the broad distribution of continuous dose variables, and the substantial fraction of patients at low risk of toxicity (as dictated by good clinical practice) limit the upper ceiling for the AUC well below the theoretical best value of 1. Further, population sizes above 1,000 patients are required to reduce the AUC confidence intervals and allow a statistically significant separation between the models with weak/medium/strong discriminative power. Bahn and Alber demonstrated that the AUC should be used with caution when modeling radiotherapy outcomes and suggest that it is prudent not to put too much store by the AUC (39). Other performance measures, such as calibration, coupled with external validation [TRIPOD type 2b or 3 (25)] should be stressed.

Our findings have implications for treating localized PCa where there is interest in dose intensification strategies and using fewer larger fractions. Both increasing doses with IMRT/VMAT and using extreme hypofractionation are likely to increase the risks of early fatigue, but other options could be considered. Since brachytherapy delivers a highly conformal dose to the clinical target volume without redistributing the dose to a larger volume of tissue, combining EBRT with a high-dose-rate brachytherapy boost would allow dose escalation to the prostate gland without increasing the ID in patients deemed at high risk for fatigue (40–42). A randomized trial by Hoskin et al. showed improved biochemical progression-free survival, although the control arm was no longer the standard of care when its results were available (40). However, several non-randomized studies reported impressive biochemical disease-free survival rates with tolerable toxicity with this approach (43, 44).

Our work also has implications for treating pelvic nodes where there is a lack of consensus among radiation oncologists on their inclusion in the clinical target volume due to conflicting results from clinical trials (43, 44). Expanding the clinical target volume to include pelvic lymph nodes would result in substantially higher IDs. Previous studies showed that radiotherapy-induced fatigue was higher in patients treated with pelvic nodal radiotherapy than in those treated with prostate-only radiotherapy (8). Since our objective was to investigate a quantitative correlation between the ID and the worsening of fatigue and functional outcomes, we excluded the patients treated with pelvic nodal radiotherapy in our study. Including these patients would have resulted in a bimodal distribution of the ID precluding a meaningful quantitative determination of its effect on the outcome. Nevertheless, in the absence of the robust evidence of efficacy, our results indicate that clinicians should consider the likelihood of increased fatigue as they weigh the risks and benefits of including pelvic lymph nodes in the clinical target volume of patients treated with radical radiotherapy.

A limitation of our study is that the outcome measure was only determined at two time points—before the commencement of radiotherapy and at the end of radiotherapy. A preliminary analysis revealed that the rates of the worsening of at least two and three functional endpoints were 23% and 12.4% at 2-year follow-up; additionally, the worsening of the functional status at the end of radiotherapy was associated with functional worsening at 2-year follow-up (OR∼3 and p-value<0.0001 for both endpoints). These findings indicate that the analysis and results focused at the end of radiotherapy are also relevant for late endpoints. Since this study was based on a secondary analysis of the data that were already collected as part of the REQUITE study, we could not use more robust tools to assess fatigue such as the Functional Assessment of Cancer Therapy: Fatigue (FACT-F) or European Organisation for Research and Treatment of Cancer Quality of Life Questionnaire-FA12 (EORTC QLQ-FA12) questionnaires (45, 46). A further aspect is associated to the choice of considering “worsening,” i.e., a difference from the baseline, in order to specifically address radiotherapy-related outcomes. The outcome measure could underestimate the effect on patients with already adverse pretreatment fatigue and functional outcome scores, especially those treated with neoadjuvant androgen deprivation. However, this possible bias should have minimal impact on the results due to the very low rate of patients with pretreatment fatigue. **Figure S6** in the **Supplementary Material** reports the distribution of scores before radiotherapy.

Further work is needed to confirm if the changes in fatigue and functional outcomes attributable to a higher ID are sustained in the long term. Nevertheless, since there is clinical equipoise in terms of the efficacy between radical surgery and radiotherapy, our results suggest that clinicians should consider increased treatment-induced fatigue and the worsening of the physical, role, and social functioning when offering external beam radiotherapy, defining clinical target volumes, and deciding treatment delivery techniques to patients with localized PCa.

## REQUITE consortium collaborators

Yolande Lievens, Department of Radiation Oncology, Ghent University Hospital, Belgium; Marc van Eijkeren, Department of Radiation Oncology, Ghent University Hospital, Belgium; Christel Monten, Department of Radiation Oncology, Ghent University Hospital, Belgium; Wilfried De Neve, Department of Radiation Oncology, Ghent University Hospital, Belgium; Stephanie Peeters, Department of Radiation Oncology, University Hospitals Leuven, Belgium; Caroline Weltens, Department of Radiation Oncology, University Hospitals Leuven, Belgium; Gilles Defraene, Department of Radiation Oncology, University Hospitals Leuven, Belgium; Erik van Limberghen, Department of Radiation Oncology, University Hospitals Leuven, Belgium; Michael Ehmann, Department of Radiation Oncology, Universitätsmedizin Mannheim, Medical Faculty Mannheim, Heidelberg University, Mannheim, Germany; Benjamin Gauter-Fleckenstein, Department of Radiation Oncology, Universitätsmedizin Mannheim, Medical Faculty Mannheim, Heidelberg University, Mannheim, Germany; Claudia Sangalli, Department of Radiation Oncology 1, Fondazione IRCCS Istituto Nazionale dei Tumori, Milan, Italy; Sara Morlino, Department of Radiation Oncology 1, Fondazione IRCCS Istituto Nazionale dei Tumori, Milan, Italy; Laura Lozza, Department of Radiation Oncology 1, Fondazione IRCCS Istituto Nazionale dei Tumori, Milan, Italy; Maria C. De Santis, Department of Radiation Oncology 1, Fondazione IRCCS Istituto Nazionale dei Tumori, Milan, Italy; Celine Bourgier, Department of Radiation Oncology, University Federation of Radiation Oncology, Montpellier Cancer Institute; Roxana Draghici, Department of Radiation Oncology, University Federation of Radiation Oncology, Montpellier Cancer Institute; Francoise Bons, ^f^ Department of Radiation Oncology, University Federation of Radiation Oncology, Institut de Cancérologie du Gard, Nimes, France; Sheryl Green, Department of Radiation Oncology, Icahn School of Medicine at Mount Sinai, New York, USA; Laura Torrado Moya, Instituto de Investigación Sanitaria de Santiago de Compostela, Spain; Department of Radiation Oncology, Complexo Hospitalario Universitario de Santiago, Santiago de Compostela, Spain; Ramon Lobato-Busto, Department of Radiation Oncology, Complexo Hospitalario Universitario de Santiago, Santiago de Compostela, Spain; Isabel Dominguez-Rios, Department of Radiation Oncology, Complexo Hospitalario Universitario de Santiago, Santiago de Compostela, Spain; Irene Fajardo-Paneque, Department of Radiation Oncology, Complexo Hospitalario Universitario de Santiago, Santiago de Compostela, Spain; Patricia Calvo-Crespo, Instituto de Investigación Sanitaria de Santiago de Compostela, Spain; Department of Radiation Oncology, Complexo Hospitalario Universitario de Santiago, Santiago de Compostela, Spain; Ana Carballo Instituto de Investigación Sanitaria de Santiago de Compostela, Spain; Department of Radiation Oncology, Complexo Hospitalario Universitario de Santiago, Santiago de Compostela, Spain; Paula Peleteiro Instituto de Investigación Sanitaria de Santiago de Compostela, Spain; Department of Radiation Oncology, Complexo Hospitalario Universitario de Santiago, Santiago de Compostela, Spain; Olivia-Fuentes-Rios Instituto de Investigación Sanitaria de Santiago de Compostela, Spain; Grupo de Medicina Xenómica (USC), Fundación Pública Galega de Medicina Xenómica, Santiago de Compostela, Spain; Irmgard Helmbold, Division of Cancer Epidemiology, German Cancer Research Center (DKFZ), Heidelberg, Germany; Erik Briers, Patient advocate, Hasselt, Belgium; Thomas Blaschke, Praxis für Strahlentherapie, Baden-Baden, Germany; Christian Weiß, Klinikum Darmstadt, Institut für Radionkologie und Strahlentherapie, Darmstadt, Germany; Christian Weißenberger, Zentrum für Strahlentherapie, Karlsruhe, Germany; Petra Stegmaier, Zentrum für Strahlentherapie, Karlsruhe, Germany; Johannes Claßen, Klinik für Strahlentherapie, St. Vincentius-Kliniken gAG, Karlsruhe, Germany; Ulrich Giesche, Klinik für Strahlentherapie, St. Vincentius-Kliniken gAG, Karlsruhe, Germany; Marie-Luise Sautter-Bihl, Klinik für Radioonkologie und Strahlentherapie, Städtisches Klinikum, Karlsruhe, Germany; Burkhard Neu, Klinik für Radioonkologie und Strahlentherapie, Städtisches Klinikum, Karlsruhe, Germany; Thomas Schnabel, Klinik für Strahlentherapie und Radiologische Onkologie, Klinikum der Stadt Ludwigshafen gGmbH, Ludwigshafen, Germany; Jörg Schäfer, Strahlentherapie Speyer, Germany; Marzia Franceschini, Department of Radiation Oncology 2, Fondazione IRCCS Istituto Nazionale dei Tumori, Milan, Italy; Gabriele Pietro, Department of Radiation Oncology, Fondazione del Piemonte per l’Oncologia Candiolo Cancer Institute, Candiolo (TO), Italy; Elena Delmastro, Department of Radiation Oncology, Fondazione del Piemonte per l’Oncologia Candiolo Cancer Institute, Candiolo (TO), Italy; Bibiana Piqué-Leiva, Radiation Oncology Department, Vall d’Hebron Hospital Universitari, Vall d’Hebron Barcelona Hospital Campus, Barcelona, Spain; Meritxel Molla, Radiation Oncology Department, Vall d’Hebron Hospital Universitari, Vall d’Hebron Barcelona Hospital Campus, Barcelona, Spain; Alexandra Giraldo, Radiation Oncology Department, Vall d’Hebron Hospital Universitari, Vall d’Hebron Barcelona Hospital Campus, Barcelona, Spain; Monica Ramos, Radiation Oncology Department, Vall d’Hebron Hospital Universitari, Vall d’Hebron Barcelona Hospital Campus, Barcelona, Spain; Victoria Harrop, Queen Elizabeth Hospital, University Hospitals Birmingham NHS Trust, Birmingham, United Kingdom; Debbie Payne, Centre for Integrated Genomic Medical Research (CIGMR), Manchester, United Kingdom; Manjusha Keni, Department of Oncology, Derby Teaching Hospitals NHS Foundation Trust, Derby, United Kingdom; Simon Wright, Department of Oncology, Leicester Royal Infirmary, University Hospitals of Leicester NHS Trust, Leicester, United Kingdom; Sridhar Thiagarajan, Department of Oncology, Leicester Royal Infirmary, University Hospitals of Leicester NHS Trust, Leicester, United Kingdom; Luis Aznar-Garcia, Department of Oncology, Leicester Royal Infirmary, University Hospitals of Leicester NHS Trust, Leicester, United Kingdom; Kiran Kancherla Department of Oncology, Leicester Royal Infirmary, University Hospitals of Leicester NHS Trust, Leicester, United Kingdom; Christopher Kent, Department of Oncology, Leicester Royal Infirmary, University Hospitals of Leicester NHS Trust, Leicester, United Kingdom; Subramaniam Vasanthan, Department of Oncology, Leicester Royal Infirmary, University Hospitals of Leicester NHS Trust, Leicester, United Kingdom; Donna Appleton, Department of Breast Surgery, Glenfield Hospital, University Hospitals of Leicester NHS Trust, Leicester, United Kingdom; Monika Kaushik, Department of Breast Surgery, Glenfield Hospital, University Hospitals of Leicester NHS Trust, Leicester, United Kingdom; Frances Kenny, Department of Breast Surgery, Glenfield Hospital, University Hospitals of Leicester NHS Trust, Leicester, United Kingdom; Hazem Khout, Department of Breast Surgery, Glenfield Hospital, University Hospitals of Leicester NHS Trust, Leicester, United Kingdom; Jaroslaw Krupa, Department of Breast Surgery, Glenfield Hospital, University Hospitals of Leicester NHS Trust, Leicester, United Kingdom; Kelly V. Lambert, Department of Breast Surgery, Glenfield Hospital, University Hospitals of Leicester NHS Trust, Leicester, United Kingdom; Simon Pilgrim, Department of Breast Surgery, Glenfield Hospital, University Hospitals of Leicester NHS Trust, Leicester, United Kingdom; Sheila Shokuhi, Department of Breast Surgery, Glenfield Hospital, University Hospitals of Leicester NHS Trust, Leicester, United Kingdom; Kalliope Valassiadou, Department of Breast Surgery, Glenfield Hospital, University Hospitals of Leicester NHS Trust, Leicester, United Kingdom; Ion Bioangiu, Department of Oncology, Leicester Royal Infirmary, University Hospitals of Leicester NHS Trust, Leicester, United Kingdom; Kufre Sampson, Department of Oncology, Leicester Royal Infirmary, University Hospitals of Leicester NHS Trust, Leicester, United Kingdom; Ahmed Osman, Department of Oncology, Leicester Royal Infirmary, University Hospitals of Leicester NHS Trust, Leicester, United Kingdom; Corinne Faivre-Finn, Division of Cancer Sciences, University of Manchester, Manchester, United Kingdom; Karen Foweraker, City Hospital, Nottingham University Hospitals NHS Trust, Nottingham, United Kingdom; Abigail Pascoe, City Hospital, Nottingham University Hospitals NHS Trust, Nottingham, United Kingdom; Claire P. Esler, City Hospital, Nottingham University Hospitals NHS Trust, Nottingham, United Kingdom; Tim Ward, Patient advocate, Pelvic Radiation Disease Association, United Kingdom; Daniel S. Higginson, Department of Radiation Oncology, Memorial Sloan Kettering Cancer Center, New York, NY, United States; Samuel Lavers, Department of Genetics and Genome Biology, Leicester Cancer Research Centre, University of Leicester, Leicester, UK

## Data availability statement

The raw data supporting the conclusions of this article will be made available by the authors, without undue reservation.

## Ethics statement

The studies involving human participants were reviewed and approved by Manchester North West UK NRES Approval 14/NW/0035. The patients/participants provided their written informed consent to participate in this study.

## Author contributions

NJ, ACh, CW, and TRan conceived the study design and wrote the first draft of the paper. ACi, TRan, and NJ analyzed the data. ACh and CW contributed to the interpretation of the data. CW is lead chief investigator and CT is deputy lead of the REQUITE study. AM, AW, PSe, CF, CC, LV, RB, VF, CT, PSy, KJ, TRat, ML, KH, GM, RE, ES, CH, MV, BA, TG, RV, DA, M-PF, MC, AV, MA-B, AG-C, PF, EG, GG, CI, VV, JC-C, CW, TRan, and ACh contributed patients to the study. AM and AW curated the database for the REQUITE study. All authors commented on and approved the final manuscript. NJ and ACi are joint first authors. ACh and TRan are joint last authors. ACi was responsible for the statistical analysis.

## Funding

REQUITE received funding from the European Union’s Seventh Framework Programme for research, technological development, and demonstration under grant agreement no. 601826. DUE-01 received funding from AIRC (Associazione Italiana per la Ricerca sul Cancro) IG 13090 and IG 16087. ACh, RE, and CW were supported by the NIHR Manchester Biomedical Research Center. ACi is supported by AIRC IG 21479. TRan was supported by Fondazione Italo Monzino, Milan. PS was supported by the ERA-NET ERA PerMed/BMBF 01KU1912. AV was supported by Spanish Instituto de Salud Carlos III (ISCIII) funding, an initiative of the Spanish Ministry of Economy and Innovation partially supported by European Regional Development FEDER Funds (INT15/00070; INT16/00154; INT17/00133; PI19/01424; PI16/00046; PI13/02030; PI10/00164), and through the Autonomous Government of Galicia (Consolidation and structuring program: IN607B). TRat is currently an NIHR Clinical Lecturer. He was previously funded by a National Institute of Health Research (NIHR) Doctoral Research Fellowship (DRF 2014-07-079). This publication represents independent research.

## Acknowledgments

We sincerely thank all patients who participated in the REQUITE study and all the REQUITE staff involved at the following hospitals: Belgium: Ghent University Hospital, Ghent and KU Leuven, Leuven; France: ICM Montpellier and CHU Nîmes; Germany: Zentrum für Strahlentherapie Freiburg; ViDia Christliche Kliniken Karlsruhe; Klinikum der Stadt Ludwigshafen gGmbH; Universitätsklinikum Mannheim. Italy: Fondazione IRCCS Istituto Nazionale dei Tumori, Milano and Candiolo Cancer Istitute—IRCCS, Candiolo; Spain: Complexo Hospitalario Universitario de Santiago, Santiago; UK: University Hospitals Leicester, Leicester and Manchester Biomedical Research Center, Manchester; USA: Mount Sinai Hospital, New York. DKFZ thanks Anusha Müller for valuable data management.

## Conflict of interest

The authors declare that the research was conducted in the absence of any commercial or financial relationships that could be construed as a potential conflict of interest.

## Publisher’s note

All claims expressed in this article are solely those of the authors and do not necessarily represent those of their affiliated organizations, or those of the publisher, the editors and the reviewers. Any product that may be evaluated in this article, or claim that may be made by its manufacturer, is not guaranteed or endorsed by the publisher.

## Author disclaimer

The views expressed are those of the authors and not necessarily those of the NHS, the NIHR, or the Department of Health.
