# Supplementary material for: High weekly integral dose and larger fraction size increase risk of fatigue and worsening of functional outcomes following radiotherapy for localized prostate cancer
Source: Front Oncol. 2022 Oct 26;12:937934. doi: 10.3389/fonc.2022.937934 (PMC9645430; doi:10.3389/fonc.2022.937934)
Supplement: Supplementary file 1 [file DataSheet_1.docx]

**Supplementary Material**

**Table S1:** The EORTC QLQ-C30 3.0 scales used in this work to define of worsening of at least 2 functional endpoints and of worsening of at least 3 functional endpoints.

|  | Version 3.0  Item numbers | Number of items |
| --- | --- | --- |
| **Functional scales** | | |
| Physical functioning | 1 to 5 | 5 |
| Role functioning | 6, 7 | 2 |
| Social functioning | 26, 27 | 2 |
| **Symptom scale** | | |
| Fatigue | 10, 12, 18 | 3 |

**Figure S1:** Selection of the REQUITE population

**
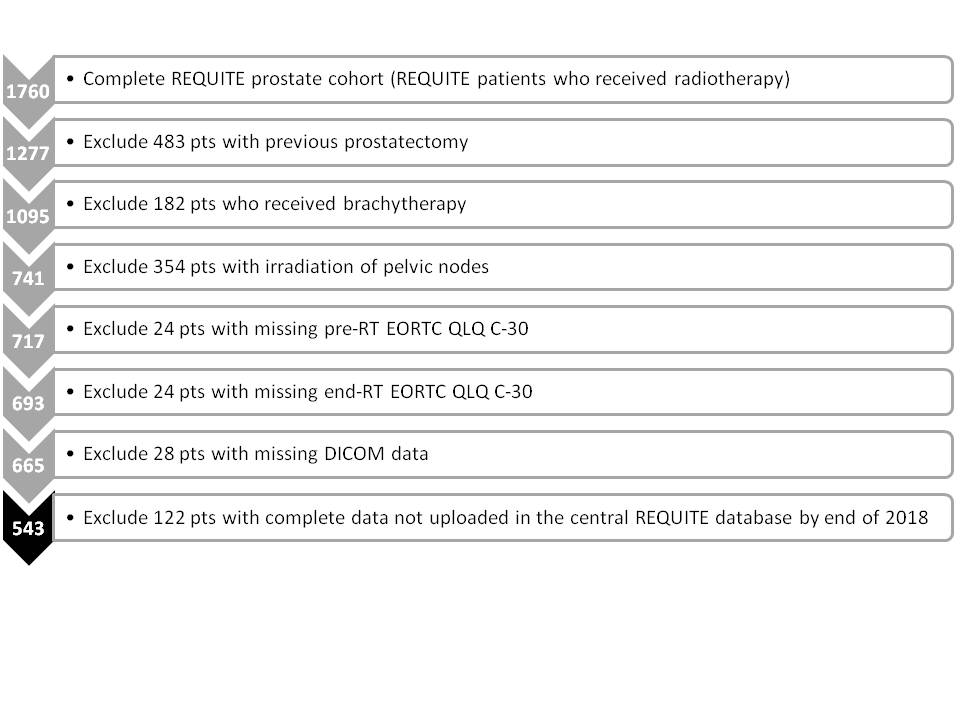
**

**Figure S2:** Selection of the DUE-01 population

**
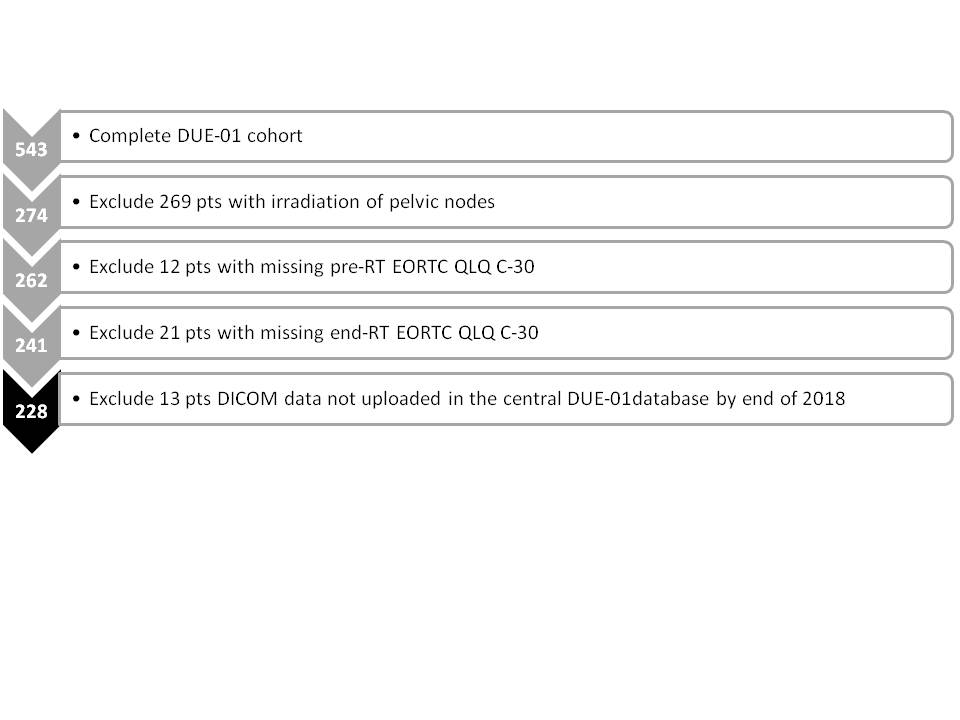
**

**Figure S3:** Rate of worsening of at least 2 functional endpoints (WS2) as a function of daily dose.

**
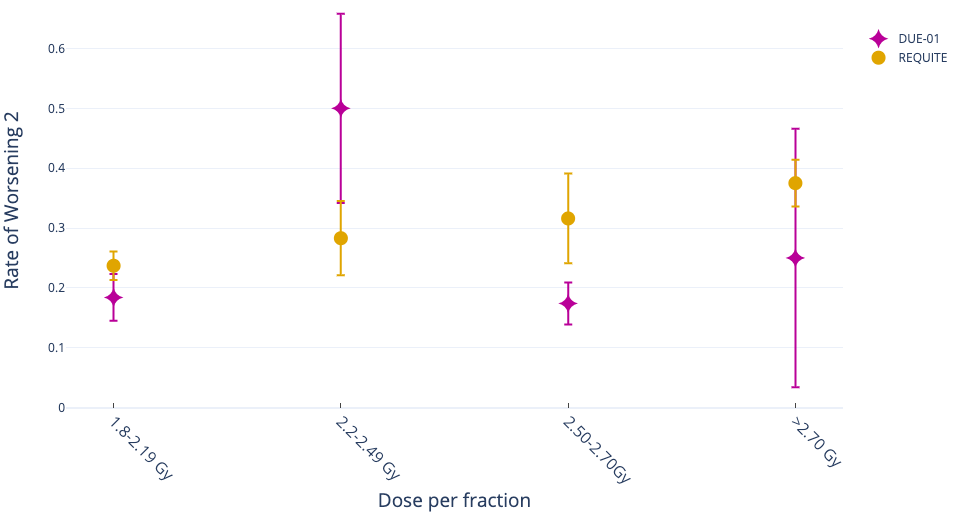
**

**Figure S4:** Rate of worsening of at least 3 functional endpoints (WS3) as a function of daily dose.


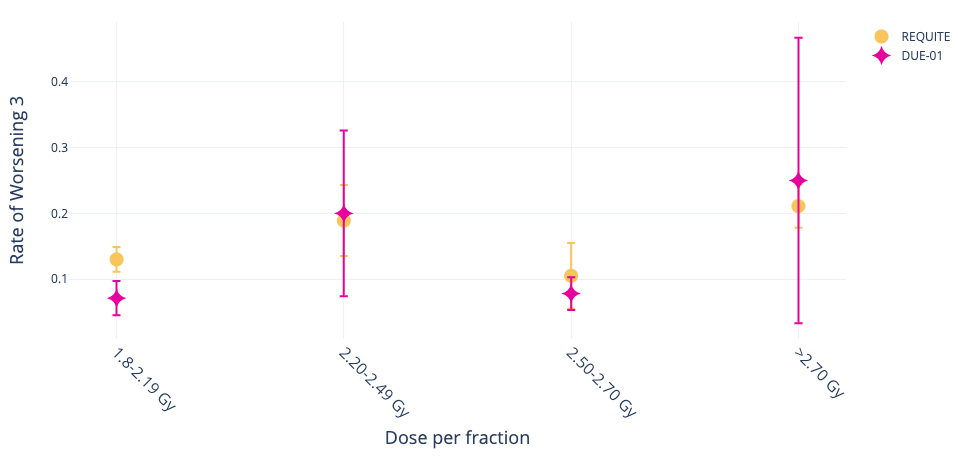


**Figure S5:** Distribution of weekly integral dose in the REQUITE cohort stratified by the radiotherapy technique (static field radiotherapy vs volumetric arc radiotherapy).


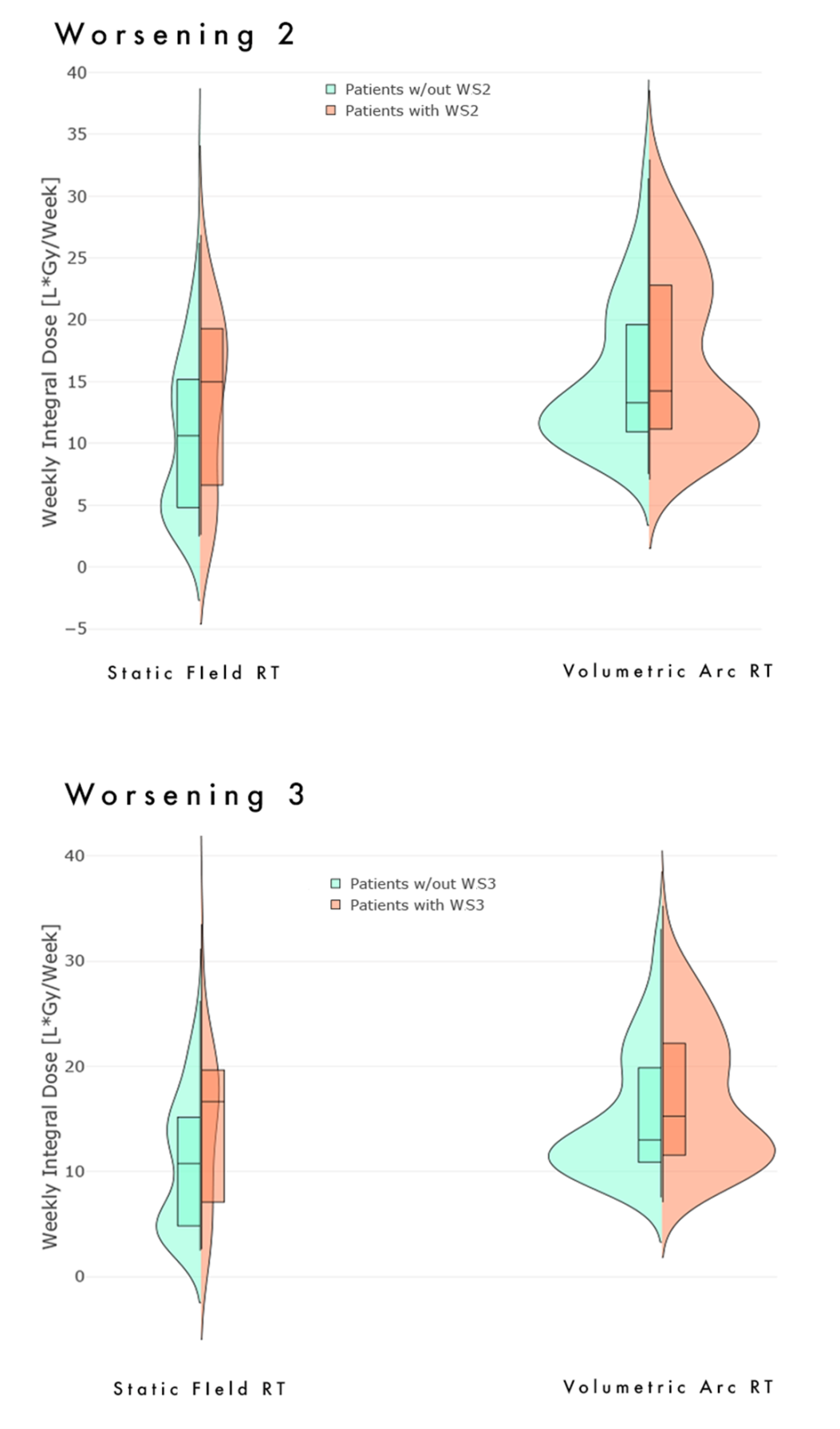


**Figure S6:** Distribution of EORTC QLQ-C30 scores considered in this work in the REQUITE and DUE-01 populations before radiotherapy, at the end of radiotherapy and at 2-year follow-up.


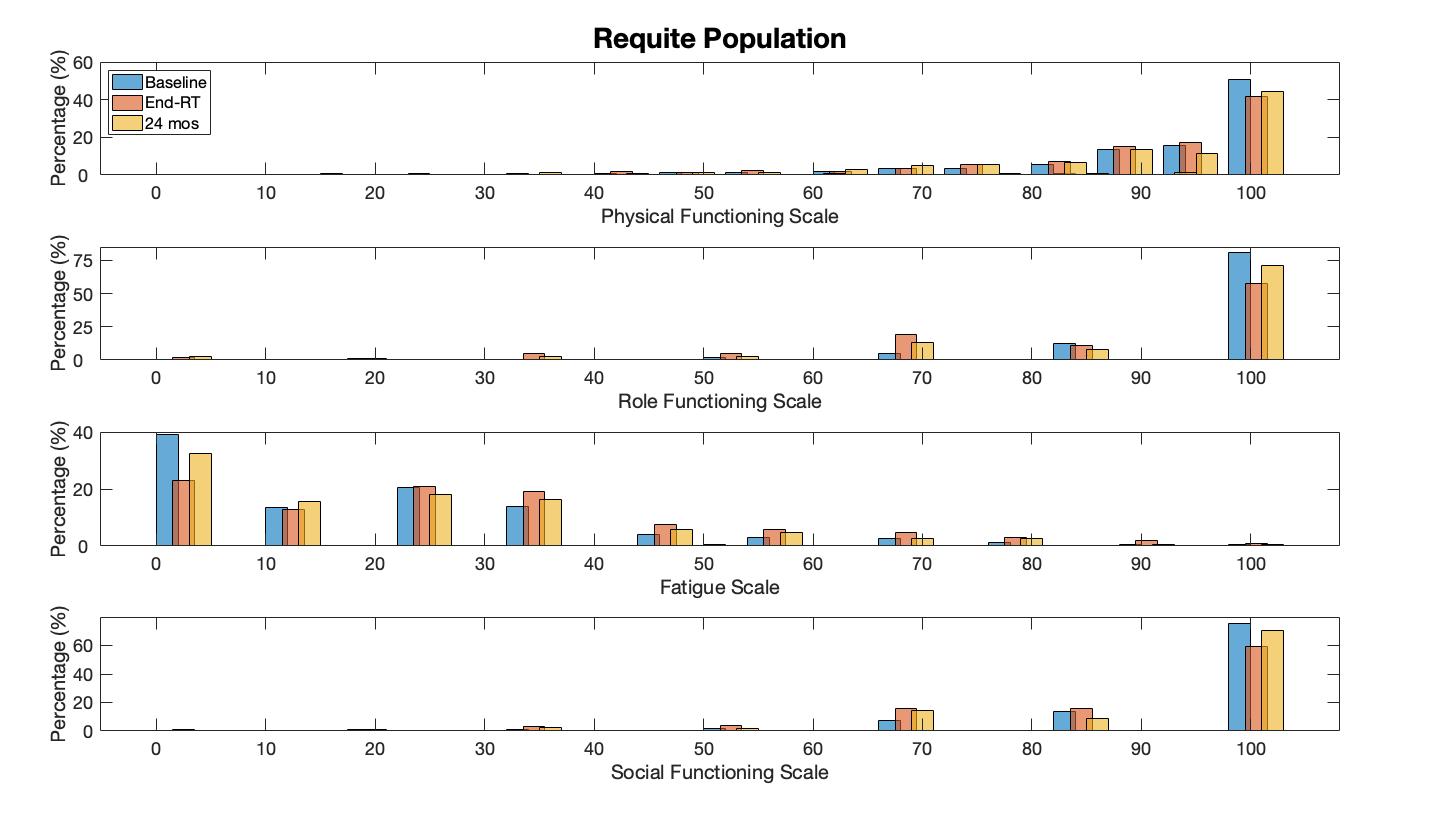


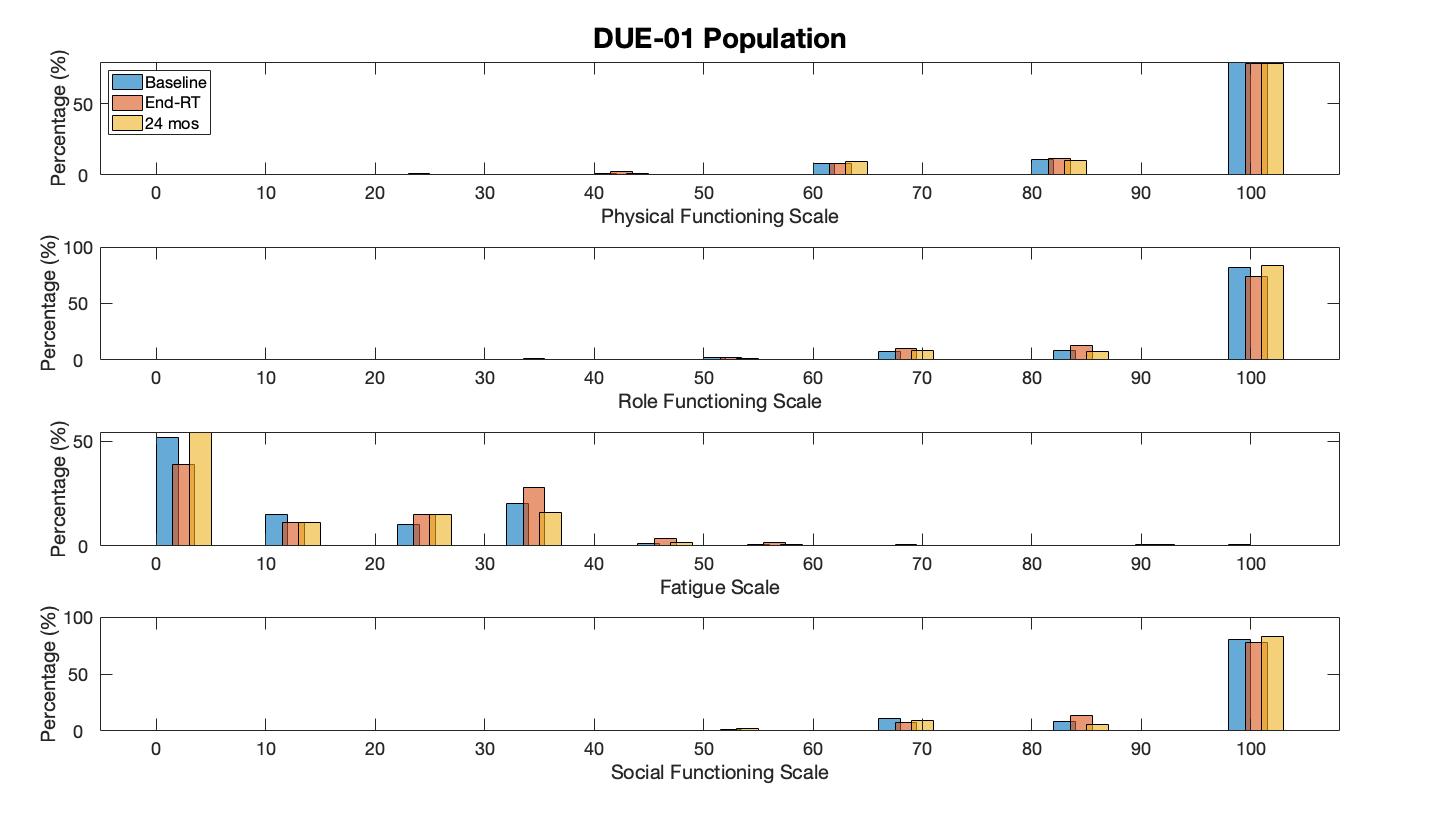


**Table S2:** Coefficients for univariate logistic models for worsening of at least 2 out 4 assessed selected EORTC QLQ 30 scales (WS2) and for worsening of at least 3 out 4 assessed EORTC QLQ 30 scales (WS3). Models for the whole population and for stratification with respect to daily dose (<2.7 Gy/fraction vs >2.7 Gy/fraction) are reported.

| **worsening of at least 2 EORTC QLQ 30 scales (WS2)** | | | | |
| --- | --- | --- | --- | --- |
|  | *Whole population* | *Treatment at dose/fr < 2.7 Gy* | *Treatment at dose/fr > 2.7 Gy* |  |
| Coefficient/OR | 0.049/1.05 | 0.039/1.03 | 0.08/1.07 |  |
| constant | -1.66 | -2.3 | -3.12 |  |
| **worsening of at least 3 EORTC QLQ 30 scales (WS3)** | | | | |
|  | Whole population | Treatment at dose/fr < 2.7 Gy | Treatment at dose/fr > 2.7 Gy |  |
| coefficient/OR | 0.053/1.06 | 0.04/1.04 | 0.086/1.09 |  |
| constant | -2.49 | -2.1 | -3.01 |  |
| *fr=fraction; OR=Odds Ratio* | | | |  |
